# Nanoparticles for Synergistic Delivery of Curcumin and Quercetin Based on Zein and Sodium Caseinate: Preparation, Characterization, and Intestinal Absorption

**DOI:** 10.3390/foods15020225

**Published:** 2026-01-08

**Authors:** Yingxi Li, Renli Shi, Zhiyue Xu, Tianyi Huang, Sitong Wang, Yaxin Sang, Marcos A. Neves, Wenlong Yu, Xianghong Wang

**Affiliations:** 1 College of Food Science and Technology, Hebei Agricultural University, Baoding 071000, China; 2 Institute of Life and Environmental Sciences, University of Tsukuba, 1-1-1 Tennodai, Tsukuba 305-8572, Ibaraki, Japan

**Keywords:** zein, curcumin, quercetin, nanoparticles

## Abstract

The purpose of the study was to characterize the basic structure of nanoparticles (Zein-CS-Cur-Que) embedded in curcumin and quercetin, realize the synergistic antioxidant of dietary polyphenols, and improve the transmembrane transport rate and bioavailability of curcumin. The oral delivery system Zein-CS-Cur-Que developed based on the synergistic encapsulation of curcumin and quercetin using the anti-solvent method with corn alkyd-soluble proteins and sodium caseinate possessed varying nanoparticle sizes (173.96–191.03 nm) and good dispersibility (PDI < 0.17), and relied on electrostatic interactions, hydrogen bonding, and hydrophobic interactions to successfully encapsulate curcumin (94.62%) and quercetin (73.75%). The results showed that Zein-CS-Cur-Que enhanced the stability and antioxidant activity of curcumin, and increased the bioaccessibility (nearly 2-fold) and rate of translocation (nearly 2-fold) of curcumin in the gastrointestinal tract significantly. Therefore, the nanocomposite system developed in this study is crucial for the development of functional foods and dietary supplements, providing effective insights into the synergy of polyphenol interactions.

## 1. Introduction

Polyphenols can be divided into flavonoids, phenolic acids, and stilbenes according to their molecular structure, and are widely distributed in fruit and vegetable diets. It is increasingly important to explore the functional properties of dietary polyphenols because of their strong biological activities [1,2]. Curcumin, as a hydrophobic polyphenol compound, is a natural active substance extracted from Curcuma plants. It has a variety of physiological and pharmacological activities, and can play a role in scavenging free radicals, antioxidation, and bacteriostasis [3,4,5]. Curcumin can also inhibit the proliferation, metastasis, and invasion of cancer cells, induce their apoptosis and autophagy, and show excellent anticancer ability [6,7,8]. However, in the actual development process, there are often problems such as low stability, poor solubility, and low bioavailability, and they are easily metabolized and eliminated by intestinal and liver enzyme systems, especially glucuronosyltransferase. Studies show that the biological activity of dietary polyphenols after synergistic action is often higher than that of a single polyphenol, and it promotes intestinal absorption to a certain extent [9,10]. Therefore, quercetin, another polyphenol, is considered for synergistic interaction with curcumin in order to realize the development and utilization of functional products, and the synergistic effect of polyphenols with an internal transport carrier promoter or external transport carrier inhibitor can enhance the ability of polyphenols to penetrate cells [11,12,13,14]. The unique anti-biofilm effect and multi-target effect of quercetin enable quercetin to reduce the rapid metabolism of curcumin by inhibiting drug-metabolizing enzymes in the synergistic process, and at the same time adjust the intestinal absorption environment to improve the stability and oxidation resistance of curcumin. Curcumin combined with quercetin can play a stronger anti-inflammatory role by inhibiting the expression of COX-2, NF-κB, and so on [15,16,17,18].

Based on the synergistic effect between polyphenols and other functional components, nanoparticles, liposomes, and micelles are used to deliver two kinds of synergistic active substances, which can not only show the synergistic effect and further improve the bioavailability, but also reduce the dosage of a single component [19,20]. Nanocarriers have been proven to be excellent materials for encapsulating phenolic compounds and improving their bioavailability. At present, the delivery system of nanoparticles based on protein is receiving unprecedented interest, and it can be used to wrap different active ingredients and achieve targeted release while reducing external influence [21,22,23]. It has advantages such as high loading rate, good biocompatibility, biodegradability, and so on. Its unique spatial structure and various amino acid residue groups make its multiple binding modes interact with functional factors, forming multiple biological functions of biocatalysis and molecular recognition [24,25].

Zein is the main storage protein in corn, and its molecule contains more than 50% hydrophobic amino acid residues, which makes it insoluble in water [26,27]. Zein can self-assemble into nanoparticles by encapsulating guest molecules through the anti-solvent method and hydrophobic interaction, and it has a wide application prospect in the field of preparing delivery carriers [28,29,30]. Because of its unique high water dispersibility and strong physical stability, sodium caseinate can modify the encapsulation and transport capacity of zein nanoparticles and enhance drug absorption and transport [31,32]. Therefore, in this study, quercetin was combined with curcumin, and a delivery carrier based on zein was constructed to co-deliver curcumin and quercetin, which not only improved the stability and absorption of curcumin in vivo, but also exerted its synergistic effect, further improved the bioavailability of curcumin, and enhanced its intestinal health functional activity.

## 2. Materials and Methods

### 2.1. Materials

Curcumin (>98%) and quercetin (>98%) were procured from Chenguang Biotechnology Group (Handan, China), while zein and sodium caseinate were sourced from Macklin (Shanghai, China). Caco-2 cells were obtained from Saiwen Innovation Biotechnology (Beijing, China). Dulbecco’s Modified Eagle’s Medium (DMEM), dimethyl sulfoxide (DMSO), and CCK-8 kit were also procured (Beijing, China), were acquired from Solepol Technology Co. Ltd (Beijing, China). Hank’s balanced salt solution (HBSS), phosphate-buffered saline (PBS), and fetal bovine serum (FBS) were obtained from Vincent Biotechnology Co., Ltd. (Nanjing, China). 6-well cell culture clusters and 96-well cell culture clusters were purchased from Guangzhou Jet Biofiltration Co., Ltd. (Guangzhou, China). Alkaline phosphatase (ALP) and bicinchoninic acid (BCA) assay kits were purchased from Nanjing Jiancheng Bioengineering (Nanjing, China).

The following instruments were utilized in this study: Model 1500-28 Enzyme Labeler (Thermo Fisher Scientific, Waltham, MA, USA), Model TGL-16G Centrifuge (Beijing Dinghao Yuan Technology Co., Ltd., Beijing, China), High-Speed Benchtop Freezing Centrifuge (Hunan Xiangyi Laboratory Development Co., Ltd., Changsha, China), Model Rotary Evaporator (Shanghai Yarong Biochemistry Instrument Factory, Shanghai, China), Model KQ-500De Ultrasonic Cleaner (Kunshan Ultrasonic Instrument Co., Ltd., Kunshan, China), Vortex Mixer (Scilogex MX-F, Rocky Hill, CT, USA), Nanoparticle size and Zeta potential analyzer (Malvern Company, Malvern, UK), Fourier transform infrared spectroscopy (Shimadzu IRXross, Kyoto, Japan), Scanning electron microscope (ZEISS Gemini SEM, Oberkochen, Germany), Specific surface area and porosity analyzer (Micromeritics, Norcross, GA, USA), Ultraviolet spectrophotometer (Shimadzu UV3600, Kyoto, Japan), X-ray diffractomer (Shimadzu XRD-7000S/L, Kyoto, Japan), fluorescence spectroscopy (Agilent Cary Eclipse; Santa Clara, CA, USA).

### 2.2. Cell Viability Testing of Individual Drugs and Synergistic Drugs

Caco-2 cells originate from human colon adenocarcinoma, but after in vitro culture, they can differentiate into the morphology and function of small intestinal epithelial cells, and then form a single-cell structure. At the same time, Caco-2 cells can express a variety of drug transporters and metabolic enzymes, and can detect the uptake, transport, and metabolism of drugs. Therefore, Caco-2 cells are selected to detect the uptake and transport of curcumin in nanoparticles.

Curcumin and quercetin were first weighed and dissolved in DMSO (<0.1% (*v*/*v*)) to form a masterbatch at a concentration of 1 mg/mL, which was filtered and sterilized, and then stored away from light. For determination of cell viability, complete medium (containing DMEM 80% (*v*/*v*), FBS 20% (*v*/*v*)) was used to dilute the mother liquor to prepare different concentrations of curcumin and quercetin for determination of cell viability of the drug alone and the co-drug. The viability of cells was detected by the CCK-8 method. After 24 h of incubation, 10 μL of CCK-8 solution was added to each well and incubated for 1 h. The absorbance of each well was measured at 450 nm on an enzyme marker to calculate the cell survival rate.

### 2.3. Synergistic Drug Uptake In Vitro

Caco-2 cells in the logarithmic growth phase were taken and planted in 6-well plates at 2 × 10^5^ cells per well; after 7 days of culture, the cell surface was washed twice with 2 mL of HBSS buffer solution per well, 2 mL of synergistic solution of curcumin and quercetin at a fixed concentration ratio was added to each well, and the cells were cultured in a thermostatic incubator for 4 h. The uptake of the lysate was terminated by pre-cooled HBSS in each well, and the residual cells were washed twice with pre-cooled PBS buffer solution to determine the protein concentration; after lysis, an equal amount of methanol was added for extraction

### 2.4. Preparation of Nanoparticles

Using zein as a carrier and sodium caseinate as a stabilizer, nanoparticles carrying curcumin and quercetin were prepared via the anti-solvent method. The zein–sodium caseinate (Zein-CS) solutions were made by mixing 10 mg/mL zein in 70% ethanol and 20 mg/mL sodium caseinate in sodium caseinate. Curcumin/quercetin in different reaction ratios (10:2, 20:4, 30:6, 40:8, 50:10, 60:12, 70:14) was added to the above mixture (Zein-CS). Following continuous stirring for 2 h, the ethanol in the nanoparticle suspension was removed using a rotary evaporator. It was then concentrated in a volumetric flask to about 25 mL. The optimum curcumin/quercetin quality ratio of 50:10 was determined for subsequent nanoparticle preparation. The final mass ratio of diverse materials within the nanoparticles was CS:Zein:Cur = 200:100:50 and CS:Zein:Cur:Que = 200:100:50:10. The former product was named Zein-CS-Cur, while the latter component was named Zein-CS-Cur-Que, which was consistent with the names of subsequent cell experiments.

### 2.5. Dynamic Light Scattering

Sample dispersions loaded with curcumin and quercetin nanoparticles with different mass ratios were diluted 10 times (*w*/*v*) with distilled water, and then 1 mL was put into a DTS0012 sample cell. Particle size and PDI were measured at 25 °C with dynamic light scattering instrument (UK, Malvern Company), the same amount of dispersion was put into a DTS1070 potential cell, and the Zeta potential of the nanoparticles was measured at 25 °C after an interval of 30 s. All samples were measured 3 times in parallel and analyzed statistically for ANOVA using SPSS 20.0. Results are expressed as mean ± standard deviation (SD). Significant differences between samples were found at 95% confidence level (*p* < 0.05).

### 2.6. Encapsulation Efficiency and Loading Efficiency

Appropriate amounts of curcumin and quercetin were weighed and dissolved in ethanol, and the absorbance values of different concentrations of the solutions at 423 nm and 325 nm were determined using a UV spectrophotometer to establish a linear relationship to obtain the standard curve. After centrifugation at 12,000 r/min, 1 mL of the supernatant was diluted ten-fold with ethanol, and the absorbance values of free curcumin and free quercetin in the diluted samples were determined [33,34]. The free curcumin and free quercetin contents in different nanoparticles were calculated from the standard curves, and encapsulation efficiency and loading efficiency were calculated according to the following equations.

(1) $$EE\left(\%\right)=\left(1-\frac{\;\mathrm{Amount}\;\mathrm{of}\;\mathrm{unencapsulated}\;Curcumin\;\mathrm{or}\;Quercetin}{\;\mathrm{Amount}\;\mathrm{of}\;\mathrm{initial}\;Curcumin\;\mathrm{or}\;Quercetin}\right)\times 100\%$$

(2) $$LE\left(\%\right)=\left(1-\frac{\;\mathrm{Amount}\;\mathrm{of}\;\mathrm{unencapsulated}\;Curcumin\;\mathrm{or}\;Quercetin}{\;\mathrm{Total}\;\mathrm{weight}\;\mathrm{of}\;\mathrm{nano}\text{-}\mathrm{particles}}\right)\times 100\%$$

### 2.7. Scanning Electron Microscope (SEM)

Take 10 mg of the sample, spray with gold for about 100 s, and uniformly disperse it on the conductive adhesive during sample making, in which the accelerating voltage is 15 KV, the working distance is 6.9 mm, and the shooting magnification is 5000–80,000, to observe the micro-morphology of nanoparticles.

### 2.8. Fourier Transform Infrared Spectroscopy (FTIR)

KBr powder was taken for individual grinding and measured using post-press as a baseline calibration; then, lyophilized nanoparticles were taken and carefully ground with KBr powder in a mortar and pestle at a mass ratio of 1:99 and again pressed into thin slices. The samples were scanned using Fourier transform infrared spectroscopy (Japan, Shimadzu IRXross); the wavelength transmittance was set to 4000–400 cm^−1^, the resolution was set to 4 cm^−1^, and the number of scans was set to 32 times. All samples were measured in parallel 3 times.

### 2.9. X-Ray Diffraction (XRD) Analysis

An X-ray diffractometer (Shimadzu XRD-7000S/L, Japan) was used to measure the diffraction effect of the crystal structure in freeze-dried samples ground to powder. The radiation source was Cukα (λ = 1.5418), the current was 30 mA, the test speed was 0.05 s per step, the scanning range was 5–80, and the number of measurements were 3 times.

### 2.10. Fluorescence Spectroscopy (FS)

The interactions between curcumin, quercetin, zein, and sodium caseinate were investigated using fluorescence spectroscopy (Agilent Cary Eclipse), and the fluorescence spectra of the composite nanoparticles were determined using deionized water for dilution; the emission spectral range was set at 450–760 nm, and the excitation wavelength was set at 425 nm. The excitation and emission slits were set at 10 nm, and the scanning speed was set at 240 nm/min.

### 2.11. Ultraviolet and Visible Spectrum (UV)

The Ultraviolet spectrophotometer (Japan, Shimadzu UV3600) was used for full-wavelength scanning to analyze the structural properties of the samples. 2 mL of the samples was diluted 20 times and loaded into a quartz spectrophotometer, and the spectra were obtained by scanning the samples at wavelengths ranging from 200 to 800 nm; the resolution was above 1 nm, and distilled water was used as a blank control.

### 2.12. Thermogravimetric Analysis (TGA)

The sample was weighed accurately with an analytical balance at 5 mg, and placed in an alumina crucible with a heating rate of 10 °C/min, a temperature range of 25–800 °C, and a nitrogen flow rate of 20 mL/min.

### 2.13. Brunauer–Emmett–Teller (BET)

A nitrogen adsorption–desorption test was carried out to determine the specific surface area and pore structure parameters. 100 mg of the sample was accurately weighed and degassed under vacuum at 120 °C for 4–6 h for the final determination of the specific surface area, pore volume, and pore size distribution.

### 2.14. DPPH, ABTS and Total Antioxidant Capacity

The antioxidant capacity of blank nanoparticles as well as curcumin nanoparticles was evaluated using DPPH, ABTS, and total antioxidant capacity. For the determination of DPPH, 200 μL of supernatant was aspirated and the absorbance value at 517 nm was measured. Anhydrous ethanol was used as a blank control and 500 μg/mL of ascorbic acid was used as a positive control. For the determination of ABTS, 100 μL of the diluted solution and 100 μL of ABTS solution were drawn up and the corresponding absorbance values were measured at 734 nm; distilled water was used as a blank control, and 500 μg/mL ascorbic acid was used as a positive control. The total antioxidant capacity (T-AOC) was measured at 593 nm according to the total antioxidant capacity (T-AOC) assay kit procedure.

### 2.15. In Vitro Digestion Simulation

7.5 mL of simulated oral solution containing 0.896 mg/mL KC1 solution, 0.298 mg/mL NaCl solution, and 0.6 mg/mL a-amylase solution was prepared and mixed with 7.5 mL of the initial sample, the pH of the system was adjusted to 7.0, and the sample was digested for 10 min. 15 mL of simulated gastric solution containing 2 mg/mL NaCl and 3.2 mg/mL pepsin was prepared and mixed with the sample after oral digestion, and the pH of the system was adjusted to 2.5; the sample was digested for 2 h. The pH of the gastrically digested sample was adjusted to 7 with 1 mol/L NaOH; 7.5 mL of simulated intestinal fluid was added with 1 mg/mL pancreatic enzyme, 20 mg/mL porcine bile salts, and 0.11 mg/mL CaCl_2_, and the pH of the system was adjusted to 7.0 for 2 h. After centrifugation at 12,000 rpm, the supernatant of the simulated in vitro digested solution was subjected to ultraviolet (UV) measurement of the absorbance value, and the curcumin content in the micellar phase was determined.

### 2.16. Transwell Analysis

Based on the synergistic drug uptake experiments and characterization experiments of the nanoparticles, the cellular experimental encapsulation concentration ratio of the nanoparticles was determined (80:40:20:4), and the cellular viability and cellular uptake were compared for curcumin alone (Cur), curcumin–quercetin (Cur-Que), Zein-CS-Cur, and Zein-CS-Cur-Que for Caco-2 cell survival and cellular uptake and modeling of Caco-2 cell monolayer permeation. Cells in the logarithmic phase were trypsin-digested and resuspended, with cell density controlled at 1 × 10^5^/mL, and inoculated into 6-well Transwell plates in a small culture chamber. 1.5 mL of cell suspension was inoculated on the top side of the filter membrane, and 2.5 mL of complete medium was added on the basolateral side of the membrane; the medium was changed every other day for the first 7 d, and changed every day after 7 d until the Caco-2 formed a compact monolayer at the end of the 21-day period. And in order to evaluate whether the cell monolayer model was successfully established, the cell growth was observed by fluorescence inverted microscope, the transmembrane resistance value of the Caco-2 cell monolayer was measured using a cytoresistivity meter, and the ALP activity of the AP and BL sides of the cells was detected by the ALP kit.

Caco-2 cells were selected for bidirectional transmembrane transport assay; the cell monolayer was washed gently with pre-warmed HBSS 3 times, 1.5 mL of HBSS was added to the upper compartment, 2.5 mL of HBSS was added to the lower compartment, and the cell monolayers were incubated for 30 min at 37 °C. After the incubation was completed for the forward transport assay (AP-BL), equal concentrations of curcumin of different groups were added to the upper compartment (Cur, Cur-Que, Zein-CS-Cur, Zein-CS-Cur-Que) in equal concentrations: 1.5 mL of curcumin (Cur, Cur-Que, Zein-CS-Cur, Zein-CS-Cur-Que) was added to the upper chamber, 2.5 mL of blank HBSS was added to the lower chamber, and the incubation was carried out for 4 h at 37 °C. Secondly, for the reverse curcumin transport (BL-AP), 1.5 mL of blank HBSS was added to the upper chamber, 2.5 mL of the sample solution was added to the lower chamber, and the same incubation was carried out at 37 °C for 4 h. Upon completion of the incubation, the sample was incubated for 30 min. After the incubation, the receiving solution was collected in the upper or lower chamber, respectively, and the curcumin content was determined by HPLC. The transfer concentration and apparent permeability coefficient of curcumin were calculated.

(3) $$P_{app}=(dC/dt)/(S\times C_{0})$$

where *dC*/*dt* is the change in curcumin concentration per unit time on the receiving side, *S* is the surface area of the cell membrane, and *C*_0_ is the concentration of curcumin in the initial sample.

(4) $$P_{\mathrm{appratio}\;}=P_{\mathrm{app}\;\mathrm{BL}\text{-}\mathrm{AP}\;}/P_{\mathrm{app}\;\mathrm{AP}\text{-}\mathrm{BL}}$$

where *P*_appratio_ is the bidirectional transport coefficient, *P*_app BL-AP_ is the reverse transport apparent osmotic coefficient, and *P*_app AP-BL_ is the forward transport apparent osmotic coefficient.

### 2.17. Statistical Analysis

Experimental data were statistically analyzed using SPSS 20.0, while correlation images were generated using GraphPad Prism version 9.0. Results are expressed as mean ± standard deviation (SD). Significant differences are indicated by the following markers: * *p* < 0.05, ** *p* < 0.01, *** *p* < 0.001, and **** *p* < 0.0001. Different letters (a–h) indicate significant differences between groups.

## 3. Results and Discussion

### 3.1. Curcumin, Quercetin, and Curcumin–Quercetin Cytotoxicity Assay

**Figure 1:**
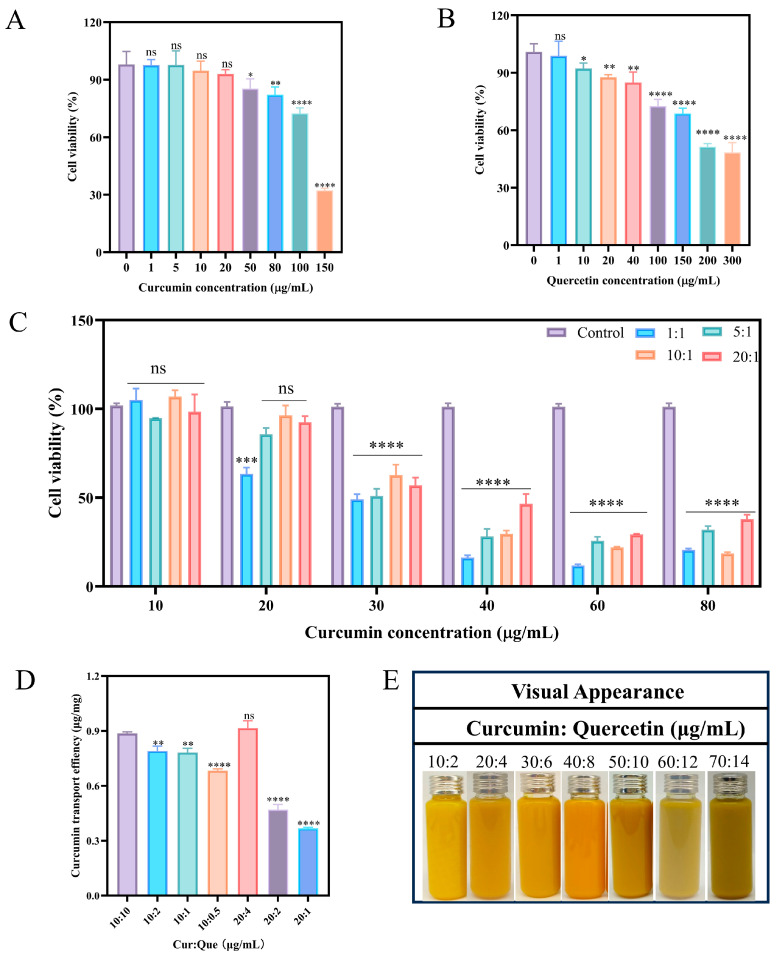
Cytotoxicity assay, cellular uptake assay, and fabrication of Zein-CS-Cur-Que. (**A**) Cytotoxicity results of curcumin at different concentrations in Caco-2 cells. (**B**) Cytotoxicity results of quercetin at different concentration in Caco-2 cells. (**C**) Cytotoxicity results of curcumin–quercetin at different concentration ratio in Caco-2 cells. (**D**) Curcumin intake of curcumin–quercetin at different concentration ratio in Caco-2 cells. (**E**) Appearance and morphology of seven different ratios (10:2, 20:4, 30:6, 40:8, 50:10, 60:12, 70:12) of Zein-CS-Cur-Que were prepared. (Data are mean and SD, n  =  3 per group, significant difference compared with control group: ns: no statistical significance, *p*  <  0.05  = *, *p*  <  0.01 = **, *p*  <  0.001 = ***, *p*  <  0.0001 = ****).

Cell viability was assayed using CCK-8 assay to evaluate the cytotoxicity of curcumin, quercetin, and curcumin–quercetin on Caco-2 cells (Figure 1A–C). The cell viability was above 80% at curcumin concentrations of 1–100 μg/mL and above 80% at quercetin concentrations of 1–40 μg/mL, and there was no significant inhibitory effect on Caco-2 cells in this concentration range, which can be applied to subsequent experiments.

After determining the tolerance concentration ranges of curcumin alone and quercetin alone for Caco-2 cells using cytotoxicity assays, cytotoxicity tests were performed with different synergistic concentration ratios of curcumin and quercetin. Curcumin concentrations of 10 μg/mL, 20 μg/mL, 30 μg/mL, 40 μg/mL, 60 μg/mL, and 80 μg/mL and quercetin ratios of 1:1, 5:1, 10:1, and 20:1 were used to determine the cytotoxicity of curcumin and quercetin, respectively. As can be seen from the result graph, after different concentrations of synergistic ratios, the cell viability was significantly reduced compared to the two alone, indicating that the two act synergistically to inhibit the proliferation of Caco-2 cells; therefore, synergistic groups with cell viability greater than 80% were selected for subsequent experiments. After comprehensive observation, it was found that the seven synergistic combinations of quercetin ratios of 1:1, 5:1, 10:1, and 20:1 at a curcumin concentration of 10 μg/mL and quercetin ratios of 5:1, 10:1, and 20:1 at a curcumin concentration of 20 μg/mL did not have any significant inhibitory effect on the Caco-2 cells, and could be used for the subsequent experiments, and the differences were statistically significant (*p* < 0.05).

### 3.2. Synergistic Drug Uptake In Vitro

Selected specific synergistic concentrations of curcumin and quercetin were subsequently evaluated for their effect on curcumin uptake by Caco-2 cells, and the biosafety of curcumin and quercetin was verified (Figure 1D). At curcumin concentrations of 10 μg/mL and 20 μg/mL, the uptake of curcumin by Caco-2 cells showed a significant increase with the increase in quercetin concentration, suggesting that the combination of curcumin and quercetin may have a synergistic effect to a certain extent, which may improve the stability and dissolution rate of curcumin and accelerate the uptake rate of curcumin by Caco-2 cells. The highest uptake rate of curcumin was 0.916 ± 0.032 μg/mg at curcumin and quercetin concentrations of 20 μg/mL and 4 μg/mL, and the synergistic ratio of the two was 5:1; this ratio (5:1) was subsequently set for the preparation of nanoparticles and characterization and analysis.

### 3.3. Dynamic Light Scattering, Encapsulation Efficiency and Loading Efficiency

**Figure 2:**
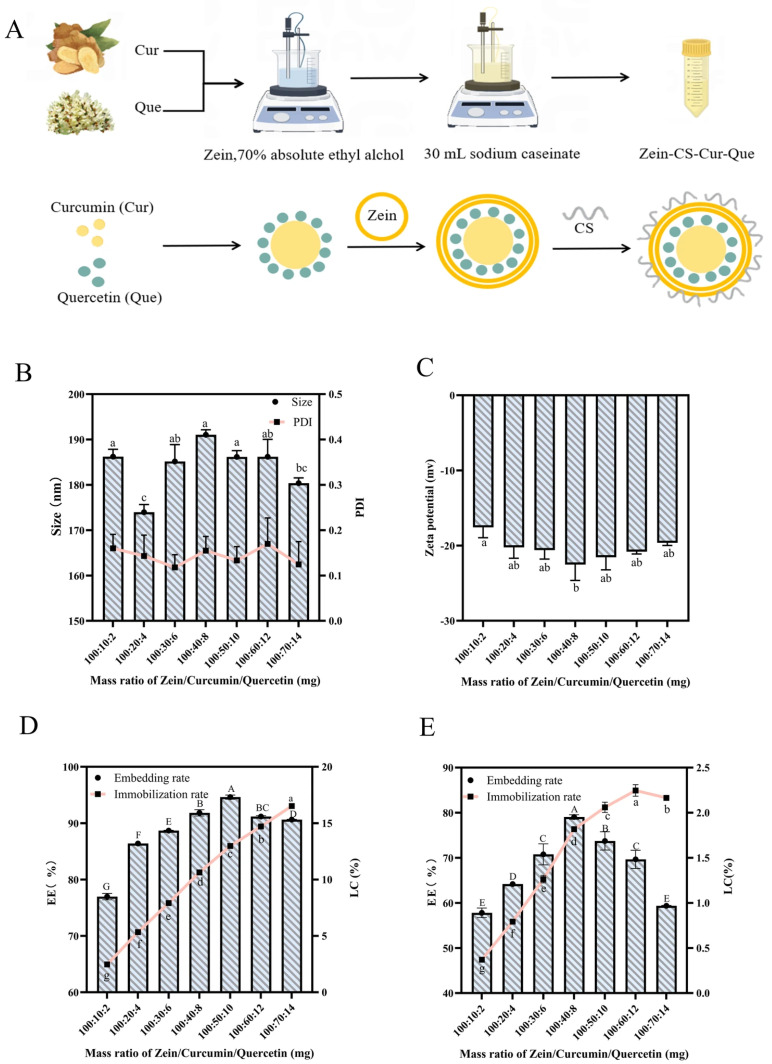
Fabrication and characterization of Zein-CS-Cur-Que. (**A**) Flow chart of the preparation process of Zein-CS-Cur-Que nanoparticles. (**B**) Mean particle and PDI of seven different ratios of Zein-CS-Cur-Que. (**C**) Zeta potential of seven different ratios of Zein-CS-Cur-Que. (**D**) Encapsulation efficiency and loading capacity of curcumin in seven different ratios of Zein-CS-Cur-Que. (**E**) Encapsulation efficiency and loading capacity of quercetin in seven different ratios of Zein-CS-Cur-Que. (Data are mean and SD, n  =  3 per group). (Different letters (a–g) and (A–G) separately indicate significant differences (*p*  <  0.05) between groups).

In order to achieve targeted release and high bioavailability of curcumin in the intestinal tract, an oral nanoparticle deliverer (Zein-CS-Cur-Que) was developed. The structure and stability of the delivery system were adjusted and optimized, and curcumin and quercetin were embedded layer by layer to increase the retention time (Figure 1E). During the experiment, curcumin and quercetin were dispersed synergistically, and quercetin was adsorbed on the surface of curcumin. Further, the anti-solvent method and magnetic stirrer were used to disperse evenly, forming a double-layer encapsulation system with zein and sodium caseinate on the outside, which realized the layer-by-layer encapsulation and stabilization system of curcumin as a whole, in order to improve the intestinal absorption of curcumin (Figure 2A).

The average particle size (173.96–191.03 nm), PDI (0.118–0.169) (Figure 2B), and Zeta potential (−17.56 ± 1.39 mV to −22.53 ± 2.11 mV) (Figure 2C) showed that the combination of both zein and sodium caseinate resulted in a uniform overall nanoparticle size and good stability through hydrophobic interactions and hydrogen bonding. At a ratio of 100:50:10, Zein-CS-Cur-Que showed higher curcumin embedding (94.62%) (Figure 2D) and higher quercetin embedding (73.75%) (Figure 2E), which was attributed to the zein and sodium caseinate relying on electrostatic adsorption to prevent curcumin and quercetin from leaking out from the inside of the particles; however, with the increase in curcumin and quercetin in the encapsulation, the encapsulation rate gradually decreased. Therefore, in this study, 100:50:10 was chosen as the optimal modification ratio of Zein-CS-Cur-Que for the preparation of the nanoparticle delivery system.

### 3.4. Scanning Electron Microscope (SEM)

**Figure 3:**
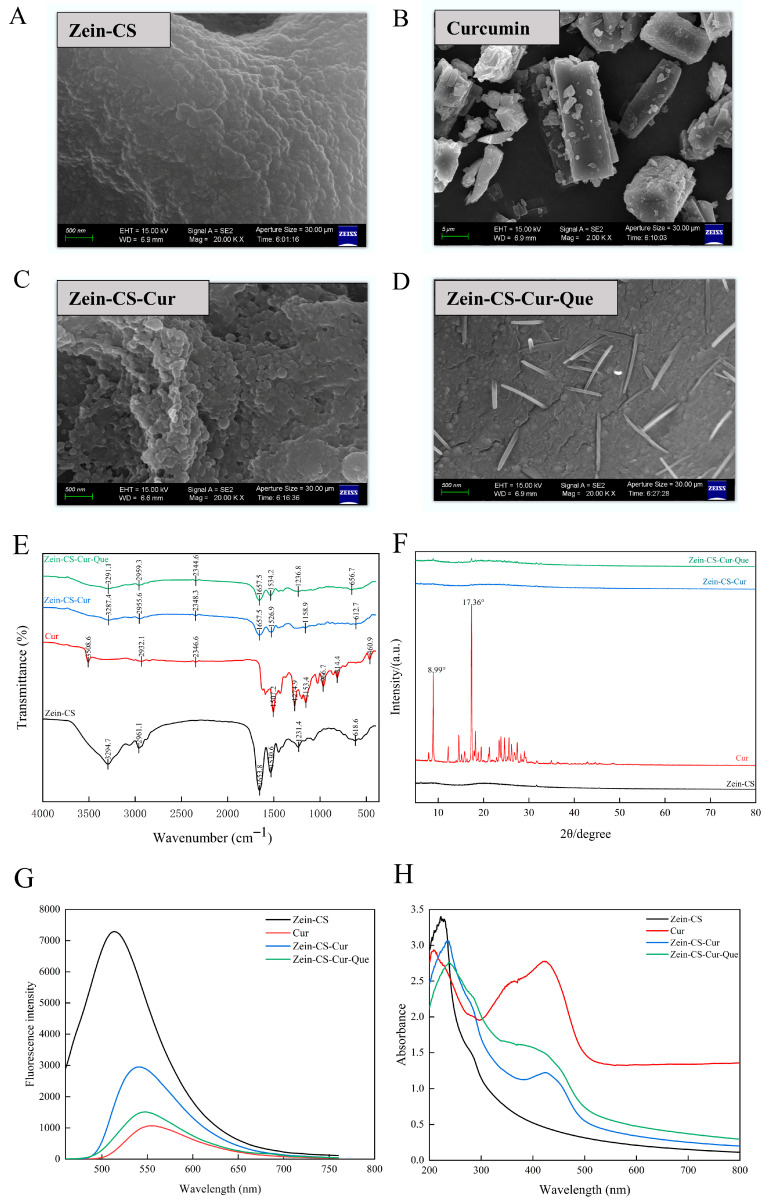
Scanning electron microscope and characterization diagram of Zein-CS-Cur-Que. (**A**) SEM images of Zein-CS. (**B**) SEM images of Cur. (**C**) SEM images of Zein-CS-Cur. (**D**) SEM images of Zein-CS-Cur-Que. (**E**) FTIR spectra of Cur and prepared nanoparticles. (**F**) XRD of Cur and prepared nanoparticles. (**G**) Fluorescence spectroscopy of Cur and prepared nanoparticles. (**H**) UV spectra of Cur and prepared nanoparticles. (Data are mean and SD, n  =  3 per).

SEM images show that Zein-CS is in a sticky state, and the nanoparticles are closely connected with each other to form an adhesive state, which is due to the strong hydrophobic interaction between zein nanoparticles and the particle aggregation phenomenon; free curcumin is a columnar or massive crystal structure, amorphous, mostly aggregated into clusters, and unevenly distributed, whereas the embedded Zein-CS-Cur can be seen to be free from the presence of curcumin crystals, with particles dispersed uniformly, and with even more severe adhesion, indicating that curcumin interacts with Zein-CS. Quercetin presents rod-shaped crystals; thus, the final Zein-CS-Cur-Que presents a self-aggregating morphology. Quercetin is partially adsorbed on the crystal surface, with a particle diameter of about 200 nm, which is in relative agreement with the results of the particle size measurement (Figure 3A–D).

### 3.5. Fourier Transform Infrared Spectroscopy (FTIR)

FTIR was used to study the interactions between the organic groups of different compounds in Zein-CS, curcumin, Zein-CS-Cur, and Zein-CS-Cur-Que (Figure 3E). The characteristic peaks of Zein-CS were 3294.7 cm^−1^ (O-H stretching vibration), 1653.8 cm^−1^ (amide I band), and 1530.6 cm^−1^ (amide II band); in the FTIR profile of free curcumin, characteristic peaks were observed at 3508.6 cm^−1^ (phenolic hydroxyl group), 1507.2 cm^−1^ (aromatic ring C-C and C-O stretching vibration), 1274.9 cm^−1^ (enol C-O stretching vibration), and 1153.4 cm^−1^ [35,36]. Compared to Zein-CS, the O-H stretching peak of Zein-CS-Cur moved from 3294.7 cm^−1^ to 3287.4 cm^−1^, indicating that hydrogen bonding was the main force in the composite nanoparticles after composite, while the two peaks in the amide I and amide II bands moved from 1653.8 cm^−1^ and 1530.6 cm^−1^, respectively, to 1657.5 cm^−1^ and 1526.9 cm^−1^, indicating that electrostatic interactions are crucial for the formation of the composite nanoparticles; the disappearance of the characteristic peaks appearing in contrast to curcumin indicates that curcumin has been successfully encapsulated [37,38,39]. And the Zein-CS-Cur-Que curcumin and quercetin peaks were slowed down after the addition of quercetin, indicating that the polyphenolic substances interacted with the proteins by relying on electrostatic interaction and hydrogen bonding, suggesting that curcumin and quercetin had been successfully encapsulated [40].

### 3.6. X-Ray Diffraction (XRD) Analysis

Curcumin has several sharp and narrow diffraction peaks, indicating a strong crystal structure characterization, while both Zein-CS-Cur and Zein-CS-Cur-Que show broad spectral bands of the polymer, and the XRD peaks exhibit a significant reduction in intensity (Figure 3F).

### 3.7. Fluorescence Spectroscopy (FS)

Using a fluorescence spectrum analyzer to analyze the interactions of Zein-CS, Cur, Zein-CS-Cur, and Zein-CS-Cur-Que (Figure 3G), it was observed that zein and sodium caseinate mainly rely on electrostatic interaction and hydrophobic interactions to maintain their stability, and strong fluorescence intensities are generated due to the presence of tyrosine and tryptophan in them. The fluorescence intensity of free curcumin was lowest in water, but after Zein-CS embedding, a significant increase in fluorescence intensity was observed, but a significant decrease in fluorescence intensity was observed with respect to untreated Zein-CS, mainly due to the fluorescence burst mechanism of the interaction between zein and curcumin, which was increased by the high level of tyrosine residues [41,42]. The addition of quercetin and curcumin promoted the fluorescence quenching of zein through molecular rearrangement, energy transduction, and collisional quenching, and thus the fluorescence intensity of Zein-CS-Cur-Que was reduced.

### 3.8. Ultraviolet and Visible Spectrum (UV)

The interactions between different compounds of Zein-CS, Cur, Zein-CS-Cur, and Zein-CS-Cur-Que were determined using a UV-Vis absorption spectroscopy analyzer with a scanning range of 200–800 nm, and subsequently analyzed (Figure 3H). The π-π jump caused by the occurrence of π-π jumps of the chromogenic groups (tyrosine, tryptophan, and tryptophan residues) present in Zein-CS caused the corresponding maximum absorption peaks measured in UV to be at 230 nm and 280 nm. Curcumin has UV absorption peaks at 207 nm and 425 nm, while the absorption peaks of Zein-CS-Cur after embedding appeared at 237 nm and 430 nm, and the intensity of the absorption peaks was significantly lower and the peaks were less frequent than that of free curcumin, indicating that the absorption peaks were red-shifted to the right, which may be caused by hydrogen bonding, hydrophobic and electrostatic interactions, and microenvironmental changes of the amino acid residues of proteins [43,44]. Quercetin had the maximum absorption peak at 341 nm, while the UV absorption peaks of Zein-CS-Cur-Que appeared at 239 nm and 368 nm, respectively. The decrease in the intensity of the characteristic peaks of curcumin and quercetin indicated the successful encapsulation, and the addition of quercetin shifted the peaks to the right, which increased the intensity of the absorption peaks as a whole.

### 3.9. Brunauer–Emmett–Teller (BET)

**Figure 4:**
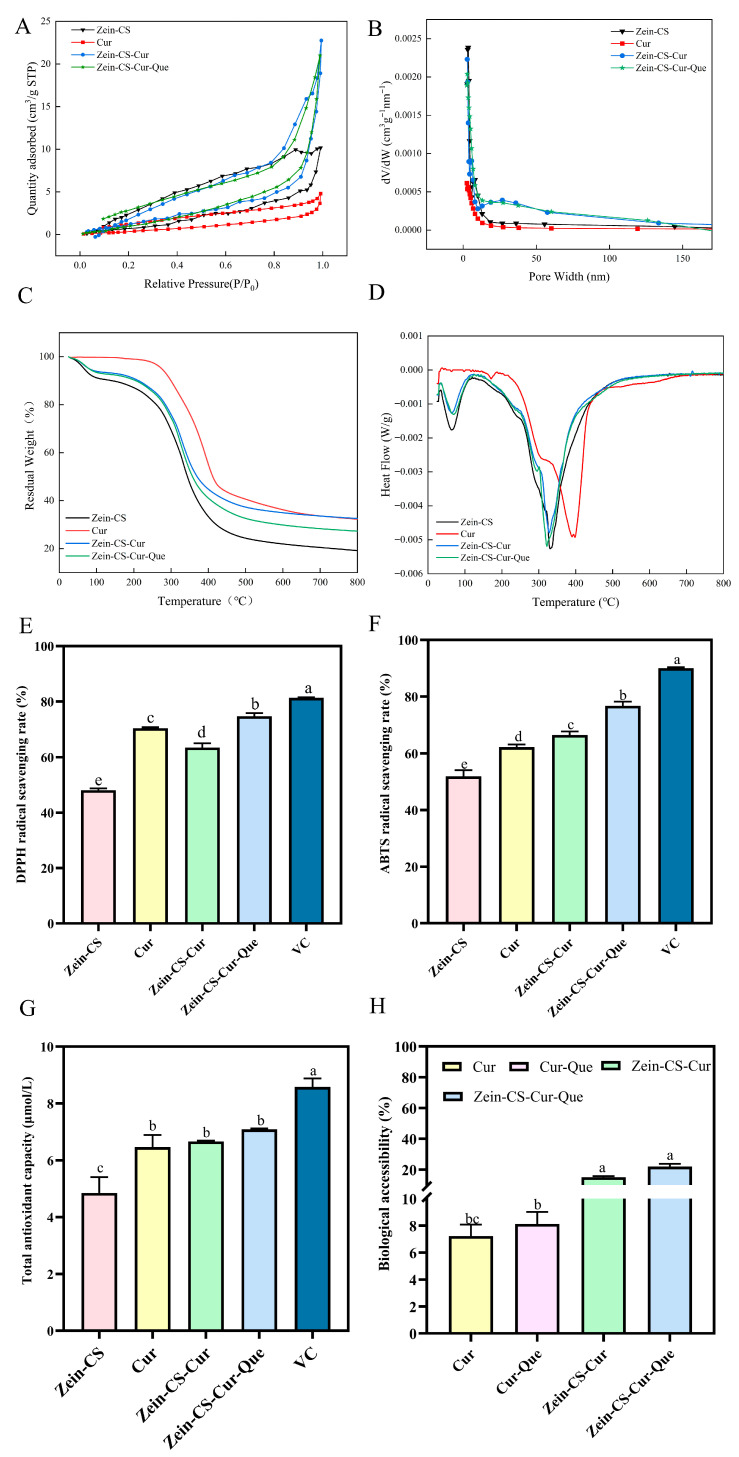
Structural characterization, antioxidant activity and bioaccessibility of Zein-CS-Cur-Que. (**A**) Isothermal adsorption curve of nitrogen sorption and (**B**) pore size distribution curve of Cur and prepared nanoparticles. (**C**,**D**) TG analysis and DTG analysis of Zein-CS, Cur, Zein-CS-Cur, and Zein-CS-Cur-Que. (**E**) DPPH clearance ratio among all groups. (**F**) ABTS clearance ratio among all groups. (**G**) Total antioxidant capacity among all groups. (**H**) Bioaccessibility of Cur in different encapsulation systems after in vitro digestion. (Data are mean and SD, n  =  3 per group, different letters (a–e) indicate significant differences (*p*  < 0.05) between groups).

BET analysis was performed to probe the specific surface area, pore volume, and pore size distribution of Zein-CS, Cur, Zein-CS-Cur, and Zein-CS-Cur-Que (Figure 4A,B). Different nanoparticles and curcumin showed Ⅳ (2–50 nm)-type adsorption isotherms with H3-type hysteresis loops, and the desorption curves showed obvious hysteresis loops at P/P0 between 0.6 and 1.0, indicating the presence of secondary mesoporous and microporous structures. It may be that curcumin is dispersed after being encapsulated by zein and sodium caseinate, forming a porous and hollow microstructure, which makes the overall specific surface area better than the original dense curcumin crystal. Zein-CS exhibited a larger specific surface area and a heel-higher pore volume after the adsorption of curcumin, which indicated that curcumin is evenly dispersed after being encapsulated by zein and sodium caseinate, forming a porous and hollow microstructure, which makes the overall specific surface area better than the original dense curcumin crystal (Supplementary Materials) [45].

### 3.10. Thermogravimetric Analysis (TGA)

When the samples were at 25 °C to 100 °C, there was a significant decrease in mass attributed to the loss of volatile compounds. The second stage, from 250 °C to 400 °C, showed a sharp decrease in mass, which was mainly due to the degradation of zein resulting in the disruption of intermolecular forces and hydrogen-bond breaking. After thermal degradation, the mass fractions of Zein-CS, Cur, Zein-CS-Cur, and Zein-CS-Cur-Que nanoparticles were 19.14%, 32.28%, 32.57%, and 27.28%, respectively, and the level of minerals present in the biopolymer samples affected the mass of the material remaining after cauterization (Figure 4C,D).

### 3.11. Antioxidant Properties and Digestion Simulation In Vitro

The stronger antioxidant activity of free curcumin relative to the control group was attributed to its phenolic hydroxyl (-OH) group that scavenges DPPH radicals and ABTS radicals (Figure 4E,F). The total antioxidant activity of encapsulated curcumin–quercetin was higher than that of the encapsulated curcumin antioxidant activity, which could be attributed to better dispersal of maize alkyd-soluble protein nanoparticles and the antioxidant properties of quercetin, resulting in better contact between curcumin and free radicals (Figure 4G).

After oral, gastric, and intestinal digestion, it is obvious that the bioaccessibility of free curcumin and Cur-Que is relatively low, at 7.21% and 8.12%, respectively, mainly due to the poor water solubility and low bioavailability of curcumin itself, and its poor solubility in acidic environment, which makes it difficult to be digested and absorbed by the gastrointestinal tract. The bioaccessibility of the curcumin group and curcumin–quercetin combination group after corn alcohol-soluble protein embedding was 15.05% and 22.02%, which indicated that the absorption of curcumin in the body could be significantly improved after embedding, and the bioaccessibility of Zein-CS-Cur-Que finally produced was about three times as much as that of free curcumin (Figure 4H).

### 3.12. Cytotoxicity Assay, Cellular Uptake, and Cell Monolayer Modeling of Zein-CS-Cur and Zein-CS-Cur-Que

**Figure 5:**
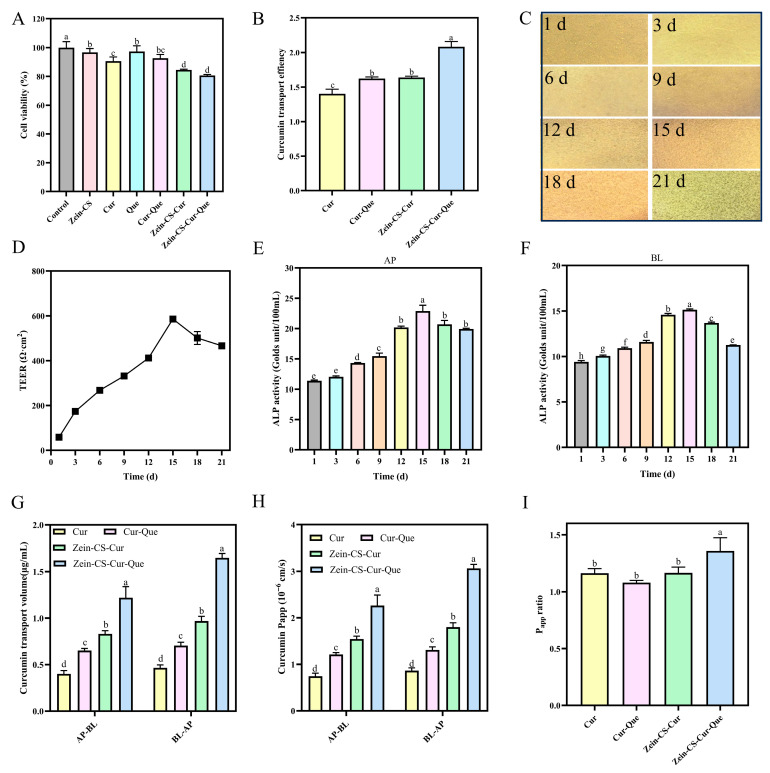
Cytotoxicity assay, cellular uptake assay, evaluation, and permeability studies of Caco-2 cell monolayer. (**A**) Cytotoxicity results of Cur and prepared nanoparticles in Caco-2 cells. (**B**) Effect of different curcumin carriers on curcumin uptake in Caco-2 cells. (**C**) Morphological observation of Caco-2 cell with different growth days. (**D**) TEER value of Caco-2 cell with different growth days. (**E**) ALP activity on the AP of Caco-2 cell with different growth days. (**F**) ALP activity on the BL of Caco-2 cell with different growth days. (**G**) Transformation of curcumin on monolayers of Caco-2 cells. (**H**) Curcumin P_app_ of different curcumin carriers. (**I**) Curcumin P_appratio_ of different curcumin carriers. (Data are mean and SD, n  =  3 per group, different letters (a–h) indicate significant differences (*p*  < 0.05) between groups).

The nanoparticle encapsulation ratio (80:40:20:5) for the establishment of cellular experiments was determined based on the pre-existing curcumin and quercetin synergistic concentration ratio (20:5) and the nanoparticle characterization ratio. The cytotoxicity of Zein-CS, Cur, Que, Cur-Que, Zein-CS-Cur, and Zein-CS-Cur-Que was determined, and the cell viability of all groups was above 80% compared to the control, which proved the good cytocompatibility; thus, it could be used for subsequent experiments (Figure 5A).

The uptake ability of curcumin and nanoparticle-embedded groups on Caco-2 cells was determined, and at a curcumin concentration of 20 μg/mL, Cur-Que, Zein-CS-Cur, and Zein-CS-Cur-Que had higher uptake ability for curcumin than the curcumin-alone group; the highest rate of uptake was observed in the final Zein-CS-Cur-Que (Figure 5B).

The artificial construction of the Caco-2 cell monolayer model was able to mimic the function and properties of small intestinal epithelial cells. Observing the cell morphology under the microscope at 21 d, the cells gradually formed a dense structure, forming a complete cell barrier; the TEER value first showed an increase and then a decrease, confirming that the cells were tightly connected, and remained above 400·Ω-cm^2^ at 21 d (Figure 5C,D). The alkaline phosphatase activities of the AP side and the BL side also first showed an increase and then a decrease, and the AP/BL ratio of the ALP viability reached more than 1.5, indicating that the monolayers had three indicators, proving that the monolayer membrane of the Caco-2 cells was successfully established and could be used for subsequent experiments (Figure 5E,F).

The P_app_ (AP-BL) of curcumin was 0.74 × 10^−6^ cm/s less than 1 × 10^−6^ cm/s, indicating malabsorption, and the permeability coefficients of the rest of the groups were significantly elevated, especially that of Zein-CS-Cur-Que at 2.26 × 10^−6^ cm/s, about more than three times that of the curcumin-alone group, suggesting that Zein-CS-Cur-Que more easily penetrated the intestinal barrier and promoted curcumin absorption [46,47]. Cur, Cur-Que, Zein-CS-Cur, and Zein-CS-Cur-Que had significantly higher P_app_ (BL-AP) than P_app_ (AP-BL) and 1 < P_appratio_ < 1.5, indicating that all four were predominantly passively transported in the Caco-2 cell monolayer model, did not have significant exocytosis, and were not substrates for exocytotic proteins (Figure 5G–I).

## 4. Conclusions

In summary, the oral delivery system Zein-CS-Cur-Que developed based on the synergistic encapsulation of curcumin and quercetin by the anti-solvent method with corn alkyd-soluble proteins and sodium caseinate possessed varying nanoparticle sizes (173.96 nm–191.03 nm) and good dispersibility (PDI < 0.17), and relied on electrostatic interactions, hydrogen bonding, and hydrophobic interactions to successfully encapsulate curcumin (94.62%) and quercetin (73.75%), which significantly improved the bioaccessibility and cellular translocation uptake of curcumin. Most importantly, the established Zein-CS-Cur-Que provides multi-pathway relief of ulcerative colitis (modulation of inflammatory factors, reduction in oxidative stress levels), restoration of the intestinal barrier (upregulation of expression levels of ZO-1, Claudin-1, and Occludin), increase in intestinal flora abundance, and promotion of short-chain fatty acid production through nutritional intervention. The nanocomposite system developed in this study is thus crucial for the development of functional foods and dietary supplements and the advancement of precision nanomedicine for ulcerative colitis.

## Data Availability

The original contributions presented in this study are included in the article. Further inquiries can be directed to the corresponding authors.

## Supplementary Materials

The following supporting information can be downloaded at https://www.mdpi.com/article/10.3390/foods15020225/s1.

## Abbreviations

Cur, curcumin; Que, quercetin; CS, caseinate sodium.
