# Supplementary material for: Nanoparticles for Synergistic Delivery of Curcumin and Quercetin Based on Zein and Sodium Caseinate: Preparation, Characterization, and Intestinal Absorption
Source: Foods. 2026 Jan 8;15(2):225. doi: 10.3390/foods15020225 (PMC12839586; doi:10.3390/foods15020225)
Supplement: Supplementary file 1 [file foods-15-00225-s001.zip › foods-4050183-supplementary.pdf]

	P/P_0	Vm	C
Zein-CS	0.0553-0.3090	0.9130	6.6041
Cur	0.0842-0.2242	0.5269	4.2029
Zein-CS-Cur	0.0573-0.3090	1.5855	6.7619
Zein-CS-Cur-Que	0.0586-0.2074	2.1809	2.1731
